# Poster Session II – Poster of Distinction II A318 UPREGULATION OF COLLAGEN 4 IS CORRELATED WITH INCREASED TISSUE STIFFNESS OF MOUSE EMBRYONIC MODEL OF HIRSCHSPRUNG DISEASE

**DOI:** 10.1093/jcag/gwaf042.318

**Published:** 2026-02-13

**Authors:** D Lee, M Zhu, S Hopyan, B Li, A Pierro

**Affiliations:** The Hospital for Sick Children, Toronto, ON, Canada; The Hospital for Sick Children, Toronto, ON, Canada; The Hospital for Sick Children, Toronto, ON, Canada; The Hospital for Sick Children, Toronto, ON, Canada; The Hospital for Sick Children, Toronto, ON, Canada

## Abstract

NOT PUBLISHED AT AUTHOR’S REQUEST

Please acknowledge all funding agencies listed below

**Funding Agencies:**

CAG, CIHRSickKids Restracomp, University of Toronto Yuet Ngor Wong

